# GITR ligand fusion protein agonist enhances the tumor antigen–specific CD8 T-cell response and leads to long-lasting memory

**DOI:** 10.1186/s40425-017-0247-0

**Published:** 2017-06-20

**Authors:** Nick M. Durham, Nick Holoweckyj, Randall S. MacGill, Kelly McGlinchey, Ching Ching Leow, Scott H. Robbins

**Affiliations:** 1 Department of Translational Medicine, MedImmune, One Medimmune Way, Gaithersburg, MD 20878 USA; 2 Department of Infectious Disease and Vaccines, MedImmune, One Medimmune Way, Gaithersburg, MD 20878 USA; 3 Department of Oncology, MedImmune, One Medimmune Way, Gaithersburg, MD 20878 USA

**Keywords:** GITRL-FP, GITR, T cell, Vaccine, CD8, Memory, Antigen-specific

## Abstract

**Background:**

The expansion of antigen-specific CD8 T cells is important in generating an effective and long-lasting immune response to tumors and viruses. Glucocorticoid-induced tumor necrosis factor receptor family-related receptor (GITR) is a co-stimulatory receptor that binds the GITR ligand (GITRL). Agonism of GITR can produce important signals that drive expansion of effector T cell populations.

**Methods:**

We explored two separate murine tumor models, CT26 and TC-1, for responsiveness to GITR Ligand Fusion Protein(GITRL-FP) monotherapy. In TC-1, GITRL-FP was also combined with concurrent administration of an E7-SLP vaccine. We evaluated tumor growth inhibition by tumor volume measurements as well as changes in CD8 T cell populations and function including cytokine production using flow cytometry. Additionally, we interrogated how these therapies resulted in tumor antigen-specific responses using MHC-I dextramer staining and antigen-specific restimulations.

**Results:**

In this study, we demonstrate that a GITR ligand fusion protein (GITRL-FP) is an effective modulator of antigen-specific CD8 T cells. In a CT26 mouse tumor model, GITRL-FP promoted expansion of antigen-specific T cells, depletion of regulatory T cells (Tregs), and generation of long-lasting CD8 T cell memory. This memory expansion was dependent on the dose of GITRL-FP and resulted in complete tumor clearance and protection from tumor rechallenge. In contrast, in TC-1 tumor–bearing mice, GITRL-FP monotherapy could not prime an antigen-specific CD8 T cell response and was unable to deplete Tregs. However, when combined with a vaccine targeting E7, treatment with GITRL-FP resulted in an augmentation of the vaccine-induced antigen-specific CD8 T cells, the depletion of Tregs, and a potent antitumor immune response. In both model systems, GITR levels on antigen-specific CD8 T cells were higher than on all other CD8 T cells, and GITRL-FP interacted directly with primed antigen-specific CD8 T cells.

**Conclusions:**

When taken together, our results demonstrate that the delivery of GITRL-FP as a therapeutic can promote anti-tumor responses in the presence of tumor-specific CD8 T cells. These findings support further study into combination partners for GITRL-FP that may augment CD8 T-cell priming as well as provide hypotheses that can be tested in human clinical trials exploring GITR agonists including GITRL-FP.

**Electronic supplementary material:**

The online version of this article (doi:10.1186/s40425-017-0247-0) contains supplementary material, which is available to authorized users.

## Background

Glucocorticoid-induced tumor necrosis factor receptor (TNFR) family-related receptor (GITR) is a co-stimulatory receptor that binds the GITR ligand (GITRL). GITR is found on CD4^+^ and CD8^+^ T cells but is most highly expressed on CD25^+^/Foxp3^+^ regulatory T cells (Tregs) [1, 2]. GITR is upregulated on T cells when activated via their T-cell receptor. GITRL is found on many types of antigen presenting cells including DCs and macrophages and GITRL can be upregulated by cytokine or TLR stimulation [3, 4]. Agonism of GITR in vitro or in vivo with its cognate ligand, agonist antibodies, or multimeric fusion proteins has been shown to increase T-cell proliferation and cytokine release [5–7]. In mouse models, levels of GITR on tumor-infiltrating T cells are significantly higher than in the periphery and are highest on tumor-infiltrating Tregs.

As a potent immune modulator, GITR signaling has been explored in tumor models to initiate or expand antitumor responses. For example, agonist molecules such as DTA-1, a Rat IgG2b antibody specific for murine GITR, have been shown to be highly effective at promoting tumor rejection in specific tumor models and promoting potent antitumor CD4 and CD8 T cell responses [8–10]. One mechanism by which DTA-1 may mediate this effect is selective inhibition or depletion of intratumoral Tregs [11–13]. GITR agonism by GITRL or agonist antibody inhibits Treg suppression function and drives non-regulatory CD4 T cells to proliferate. In addition to CD4 Treg modulation, GITR agonism has been shown to directly affect CD8 T cells by impacting responsiveness to CD28 stimulation, leading to differential levels of the mitochondrial transmembrane molecule Bcl-xL [7]. It has also been shown that CD8 T cells that upregulate GITR become interleukin-15 (IL-15)–dependent CD8 memory cells [14, 15].

Herein we explore the impact of therapeutically targeting GITR on the generation and development of tumor-specific CD8 T cells utilizing a GITR ligand fusion protein (GITRL-FP). A recent publication details the generation of the fusion protein [16], but in brief GITRL-FP is a tetrameric fusion protein comprising multimers of the extracellular domain of GITRL joined with the FC region of a murine IgG2a. First we tested the ability of GITRL-FP to impact tumor growth in a self-priming CT26 model and the dependence of CD8 T cells on therapeutic activity in this setting. Next we evaluated whether GITRL-FP could directly bind to CD8 T cells and we observed the GITRL-FP driven pharmacodynamic changes in the CD8 T cell compartment including those of tumor specific cells. Finally we explored how GITRL-FP combines with a CD8 T cell priming vaccine in the non self-priming TC-1 tumor model.

## Methods

### Animals

TC-1 experiments used female C57BL/6 mice obtained from Jackson Laboratory (catalog no. 000664; Bar Harbor, ME). CT26 experiments used female BALB/c mice obtained from Envigo (Frederick, MD). Mice were between 6 and 8 weeks of age at the time of tumor implantation. All animal experiments were conducted in accordance with guidelines established by MedImmune’s Institutional Animal Care and Use Committee (IACUC).

### Cell lines and reagents

The CT26 tumor line was obtained from ATCC and was maintained in RPMI 1640 medium supplemented with 10% fetal bovine serum. The TC-1 tumor line was obtained from American Type Culture Collection (ATCC) (catalog no. CRL 2785, Manassas, VA) and maintained in Dulbecco modified Eagle medium with 10% fetal bovine serum and 1% penicillin-streptomycin. DTA-1 and isotype antibodies were obtained from Bio X Cell (West Lebanon, NH). GITRL-FP was produced by MedImmune (Gaithersburg, MD).

### E7 SLP and vaccination

A synthetic long peptide (SLP) consisting of the 45-mer human papillomavirus 16-E7 sequence SSEEEDEIDGPAGQAEPDRAHYNIVTFCCKCDSTLRLCVQSTHVD was synthesized from New England Peptide (Gardner, MA). E7 SLP was administered at 10 or 3.3 μg and was formulated with 20 μg of deoxycytosine–deoxyguanosine (CpG) oligodeoxynucleotides 2395 (TriLink, San Diego, CA) in AddaVax (Life Technologies, Carlsbad, CA) and phosphate-buffered saline in a total volume of 50 μL. Vaccinations were administered subcutaneously into the dorsal surface of the base of the tail.

### Tumor models

For TC-1 tumor implantation, 2 x 10^4^ viable TC-1 cells were implanted subcutaneously into the left hind footpad of C57BL/6 mice. For CT26 tumor implantation, 5 × 10^5^ cells were implanted subcutaneously (SC) in the right flank of BALB/c mice. Tumor growth was evaluated by direct measurement with calipers [17]. Bidirectional measurements were collected every 2 to 4 days, and tumor volume was calculated as volume = (length · width^2^)/2.

Tumors were allowed to develop for 6 to 14 days. Mice were randomized to control and treatment groups either by weight or by tumor volume of 0.035 to 0.200 cm^3^. Mice were euthanized when the volume of the primary tumor exceeded 1-cm^3^ in mice with TC-1 footpad tumors and 2 cm^3^ in those with CT26 flank tumors, in accordance with IACUC protocol. For pharmacodynamic (PD) studies, mice were euthanized and tumors and spleens were harvested, crushed through a 70-μM filter (Corning, Corning, NY), and processed into a single-cell suspension.

### Functional T-cell responses and flow cytometry

For antigen-specific stimulations, 10^6^ to 2 × 10^6^ live cells per well were plated with 10 μg of AH1 peptide sequence SPSYVYHQF (AnaSpec, Fremont, CA) or 1 μg of E7 peptide sequence RAHYNIVTF (AnaSpec) and protein transport inhibitor (eBioscience, Santa Clara, CA). After 5 h, cells were stained in the following order: (1) Live/Dead Fixable Blue Dead Cell Stain Kit (Life Technologies), (2) extracellular proteins, (3) Foxp3 Transcription Factor Fixation/Permeabilization Concentrate and Diluent (eBioscience), and (4) intracellular cytokines. Antibodies were purchased from Biolegend, and the clones used were CD45 (clone 30-F11), CD4 (clone RM4-5), CD8 (clone 53-6.7), GITR (clone DTA-1), interferon gamma (IFNγ) (clone XMG 1.2), tumor necrosis factor alpha (TNFα), (clone MP6-XT22), Foxp3 (clone FJK-16 s), and KI-67 (clone SolA15). H2-Db E7 dextramer loaded with RAHYNIVTF was obtained from Immudex (Fairfax, VA), and the manufacturer’s protocol was followed for staining. Samples were run on either an LSR II or Fortessa flow cytometer (Becton Dickinson, San Jose, CA). All data were analyzed using FlowJo software (Treestar, Ashland, OR).

## Results

### GITRL-FP agonism of GITR in CT26 tumors

**Fig. 1:**
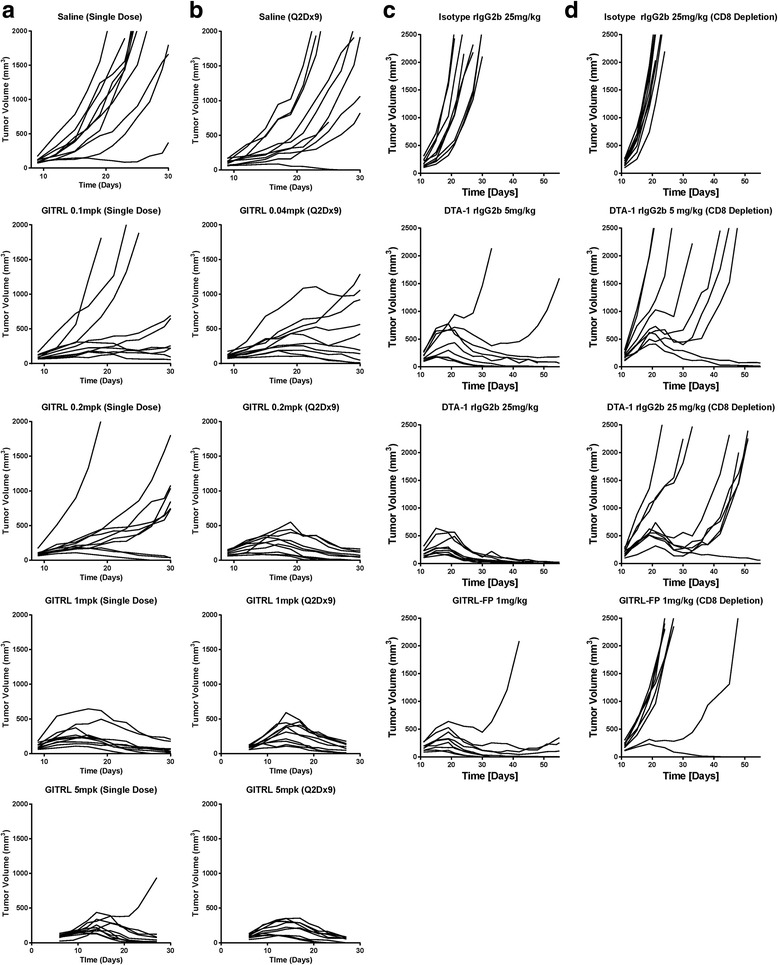
Tumor growth of CT26 tumor–bearing mice receiving IP doses of GITRL (mouse IgG2a) in (**a**) single doses or (**b**) repeated doses every other day for 9 doses. Data shown are representative of 2 repeat experiments. **c** Comparison of tumor growth in groups (*n* = 9) of CD8-depleted and nondepleted mice given IP doses of GITRL-FP or DTA-1. **d** Tumor growth rate of mice depleted of CD8 T-cells and treated with DTA-1 or GITRL-FP

To evaluate the effects of GITRL-FP on CT26 tumors, BALB/c mice were implanted with CT26 tumor cells, and on day 6 those with tumor volumes of 0.080 to 0.120 cm^3^ were randomized into groups (*n* = 10) of controls receiving saline and treatment groups receiving single or repeated administrations of GITRL-FP (mouse immunoglobulin G2a [IgG2a]). Single administrations of GITRL-FP were given at 5.00, 1.00, 0.20, and 0.10 mg/kg, and repeated administrations were given at 5.00, 1.00, 0.20, and 0.04 mg/kg biweekly for 9 total doses. Single administrations of GITRL-FP at 5 and 1 mg/kg resulted in prevention of tumor outgrowth or complete regression of CT26 tumors up to day 30 in all but one mouse (Fig. 1a). Doses of 0.2 and 0.1 mg/kg yielded 3 eliminations in the 0.2-mg/kg groups and 2 eliminations in the 0.1-mg/kg groups. Repeated administrations of GITRL-FP resulted in increased efficacy and complete regression in all but the lowest (0.04-mg/kg) dose group (Fig. 1b). The group receiving repeated administrations of 0.04 mg/kg yielded 1 elimination and 3 tumors with decreasing volumes.

To evaluate the function of GITRL-FP, we compared it with DTA-1, a known monoclonal GITR agonist antibody. DTA-1 doses were based on previous reports in which doses ranged from 5 to 25 mg/kg [10, 12, 18]. BALB/c mice were implanted with CT26 tumor cells, and on day 11 those with tumor volumes of 0.125 to 0.200 cm^3^ were randomized into groups (*n* = 9) of controls receiving saline and treatment groups receiving single administrations of GITRL-FP at 1 mg/kg or DTA-1 at 5 or 25 mg/kg (Fig. 1c). Although the efficacy of DTA-1 increased with increasing doses, the efficacy and tumor growth kinetics of GITRL-FP at 1 mg/kg were similar to those of DTA-1 at 5 mg/kg.

To determine whether CD8 T cells were necessary for the effect of GITRL-FP, we evaluated the groups treated with DTA-1 and GITRL-FP but selectively depleted CD8 T cells with a monoclonal depletion antibody on days 8, 10, 12, 14, and 16. On day 11 mice with tumor volumes of 0.125 to 0.200 cm^3^ were randomized into groups (*n* = 10) of isotype controls and treatment groups receiving DTA-1 or GITRL-FP (Fig. 1d). Without CD8 T cells, the ability of DTA-1 and GITRL-FP to completely eliminate CT26 tumors was significantly diminished: Tumor-free survival was achieved in only 1 to 2 mice from each group up to 60 days. Moreover, without CD8 T cells, GITRL-FP was less effective than DTA-1 at increasing median overall survival (Additional file 1: Table S1). To understand if mice that responded had differential CD8 depletion, we bled the mice and no differences in CD8 depletion were observed.

To understand how GITRL-FP interacts with CD8 T cells, we evaluated GITR expression on individual lymphoid populations in the same groups. On day 18, untreated mice with CT26 tumors were sacrificed to examine GITR expression on CD8 T cells and Tregs in spleens and tumors. CD4^+^ Tregs expressed higher levels of GITR than did CD8 T cells; however, both Tregs and CD8 T cells in tumors expressed higher levels of GITR than did their respective populations in spleens (Additional file 2: Figure S1).

### GITRL-FP mediation of pharmacodynamic effects with CT26

**Fig. 2:**
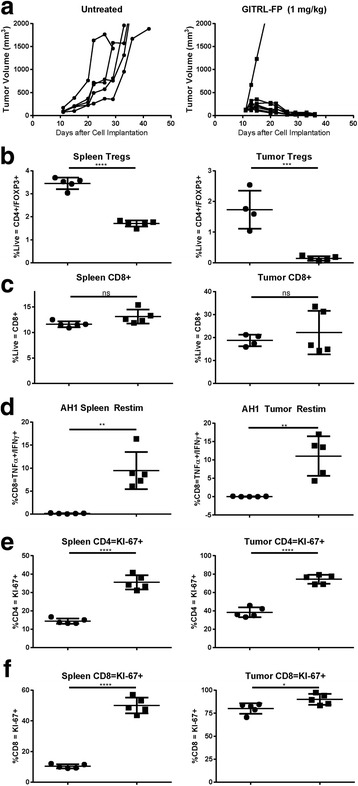
GITRL-FP PD effects on T cells in CT26 tumor–bearing mice. **a** Tumor volumes were measured after IP doses of untreated[solid *circle*●] (controls) or GITRL-FP[solid *square*■] at 1 mg/kg biweekly for 4 total doses. On day 18 mice were sacrificed to examine (**b**) Tregs and (**c**) CD8 T cells. **d** Single-cell suspensions of spleens and tumors were restimulated with AH1 peptide/protein transport inhibitor and stained for IFNγ and TNFα. Proliferation was measured by KI-67 on (**e**) CD4 T cells and (**f**) CD8 T cells

GITR agonists have been shown to decrease Tregs and to increase high-avidity T-cell responses [8, 18]. To further understand GITRL-FP mediation of pharmacodynamic effects, we evaluated the CT26 model during tumor regression. BALB/c mice were implanted with CT26 tumor cells, and on day 10 those with tumor volumes of 0.125 to 0.200 cm^3^ were randomized into untreated control groups and treatment groups receiving GITRL-FP biweekly for a total of 4 doses. The study comprised 2 arms, a tumor growth inhibition (TGI) group (*n* = 5) and a pharmacodynamic(PD) group (*n* = 5). For the PD arm, spleens and tumors were harvested on day 18 after tumor implantation. In the TGI group, complete CT26 tumor regression occurred in all but a single mouse treated with GITRL-FP (Fig. 2a).

Phenotypes of immune cells including CD8 T-cells and Tregs present in spleens and tumors were evaluated in the PD group. CD8 T-cell function was also investigated by restimulation of spleens and tumors with AH1, the CT26 immunodominant epitope, and stained for IFNγ and TNFα cytokines. GITRL-FP significantly decreased the percentage of Tregs in spleens and tumors relative to total lymphocytes (Fig. 2b). Although GITRL-FP did not change the percentage of CD8 T-cells, it significantly increased the number of CD8 T cells that were specific for AH1 (Fig. 2c and d). The percentage of total CD8s that were antigen specific and capable of producing IFNγ and TNFα was 5 to 15% in spleens and 5 to 15% in tumors, respectively, indicating significant expansion of antigen-specific lymphocytes in the tumor and the peripheral reservoir.

To evaluate whether GITRL-FP had bound to both CD4 Tregs and CD8 T cells, we stained for GITR with the DTA-1 antibody (Additional file 2: Figure S1B, C). Not surprisingly, Tregs showed more than a 75% reduction in GITR median fluorescence intensity (MFI). This could be due to downregulation of GITR on the cells or blockade of staining due to GITRL-FP occupancy. This effect was also observed on the CD8 T cells, suggesting that GITRL-FP alters DTA-1 binding on CD8 T cells. We hypothesized that, if GITRL-FP binds to GITR, T cells would show increased KI-67 proliferation similar to that reported in GITR knockout mice [19]. On day 8 after treatment, both CD4 and CD8 T cells showed significantly more KI-67–positive cells in both tumors and spleens [Fig. 2e and f].

### GITRL-FP binding of CD8 T cells in vivo

**Fig. 3:**
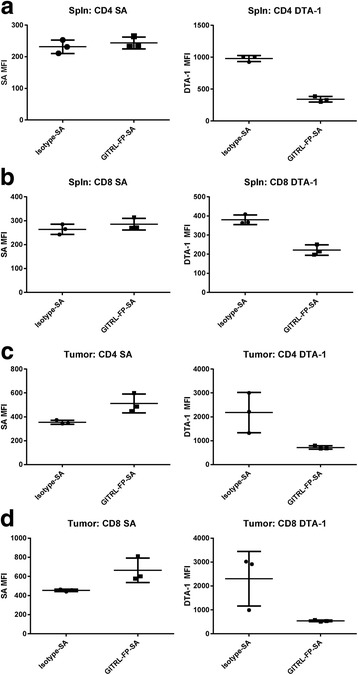
Levels of GITRL-FP bound to T-cell populations in mice receiving 3 doses of biotinylated isotype antibody or biotinylated GITRL-FP on days 11, 14, and 18. GITRL-FP drug levels were detected using SA-APC and free GITR was detected using DTA-1 anti-GITR antibody on **a**) Spleen CD4 **b**) Spleen CD8 **c**) Tumor CD4 **d**) Tumor CD8

To identify the cells with which GITRL-FP was directly interacting, we biotinylated GITRL-FP and measured the levels of labeled GITRL-FP bound to different cell populations in vivo. BALB/c mice were implanted with CT26 tumor cells, and on day 10 those with tumor volumes of 0.125 to 0.200 cm^3^ were randomized into untreated control groups and treatment groups receiving biotinylated GITRL-FP or isotype antibody on days 11, 14, and 18. Levels of labeled GITRL-FP and free GITR on the cells were measured on day 19, one day after the third dose. Streptavidin-APC (SA-APC) [Biolegend] was used as a detection reagent to evaluate the levels of drug still bound, and DTA-1 was sequentially stained to evaluate the levels of free GITR [Fig. 3a-b]. GITRL-FP–treated CD4 and CD8 T cells showed greater SA-APC MFI than did isotype-treated cells, suggesting that GITRL-FP was binding both CD4 and CD8 T cells. Although no significant difference in SA-APC was observed in the spleen, there was a significant decrease in available GITR as detected by DTA-1 in both spleens and tumors.

### GITRL-FP expansion of antigen-specific CD8 T cells

**Fig. 4:**
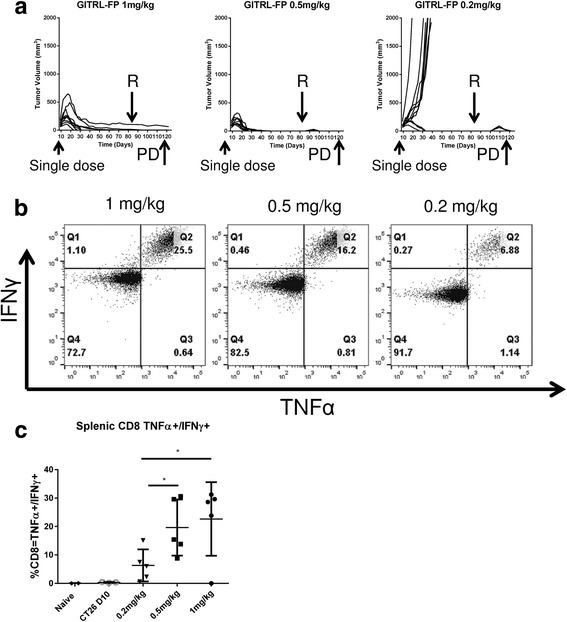
Dose-dependent GITRL-FP expansion of antigen-specific T cells. **a** CT26 tumor–bearing mice were treated with a single dose of GITRL-FP had no detectable tumors and were protected from rechallenge on day 85 (*R arrow*). **b** On day 120 after rechallenge with CT26, mouse spleens were harvested (*PD arrow*), processed to single-cell suspensions, and restimulated with 10 μg/mL AH1 peptide/protein transport inhibitor for 5 h. A dose-dependent increase was observed in AH1-specific T cells. **c** Representative plot of 5 mice from each group. For comparison, data from naïve mice and untreated mice with CT26 tumors at day 10 are shown

To determine whether mice with complete CT26 tumor regression were protected from rechallenge with the same tumor, we rechallenged CT26 tumor–bearing mice from those treated with single doses of GITRL-FP at 1.0, 0.5, or 0.2 mg/kg (Fig. 4a). All mice that originally had complete CT26 tumor regression continued to demonstrate durable protection from rechallenge with CT26 on day 85, although in the groups receiving the lowest doses (0.5 and 0.2 mg/kg), a small mass was measured that was quickly eliminated. To understand this observation, on day 120, our pharmacodynamic(PD) timepoint, we harvested spleens and restimulated splenocytes with AH1 peptide. A dose-dependent increase was seen in the numbers of AH1-specific T cells (Fig. 4b and c). In the 1-mg/kg GITRL-FP group, 25% of splenic CD8 T cells were specific for the immunodominant epitope of CT26. These mice showed no increases in tumor sizes during rechallenge. The group receiving the lowest dose, 0.2 mg/kg, had 6% antigen-specific CD8s and a small, growing mass upon rechallenge that was cleared by day 120.

### GITRL-FP generation of antigen-specific cells

CT26 is a highly immunogenic tumor model with high levels of intratumoral CD45 and basal cytotoxic T lymphocytes [20]. Although CT26 has been shown to self-prime low levels of AH1-specific T cells, this basal response is not enough to cause tumor rejection [21]. To determine whether GITRL-FP could lead to the de novo generation of antigen-specific responses, we used the E6/E7 transformed TC-1 tumor model [22]. This model has known CD8 T-cell epitopes, and previously it has been shown that the E7-specific immune response can lead to protection from tumors carrying E7 [23–25].

**Fig. 5:**
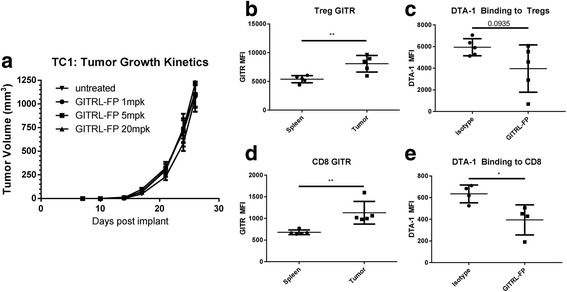
GITRL-FP effects on TC-1 tumor growth and generation of antitumor immune response in the absence of antigen-specific CD8 T cells. **a** TC-1 tumor–bearing mice received IP doses of GITRL-FP biweekly for 4 total doses. On day 24, untreated mice were sacrificed to examine (**b**) GITR expression on Tregs and (**c**) GITR expression on CD8 T cells. DTA-1 staining on (**d**) Tregs and (**e**) CD8 T cells following GITRL-FP treatment

C57BL/6 mice were implanted with TC-1 tumor cells, and on day 7 those with body weights of 0.017 to 0.020 kg were randomized into groups (*n* = 10) of untreated controls and treatment groups receiving GITRL-FP at 1, 5, and 20 mg/kg biweekly for a total of 4 doses (Fig. 5a). No delay or inhibition of tumor growth was observed at any of the doses tested. On day 24, spleens and tumors were harvested from subsets of controls and mice treated with 1 mg/kg to assess T-cell populations and E7 antigen-specific responses. No changes in CD4 or CD8 T cells were observed, and no E7-specific T cells were detected by either E7 restimulation or dextramer (data not shown). GITR levels were present on CD4 and CD8 T cells and were significantly higher in tumors (Fig. 5b and c). No measureable GITR was found on the TC-1 tumor cells using flow cytometry (data not shown). Additionally we looked to see if GITRL-FP treatment modulated the ability for DTA-1 to bind either Tregs or CD8 T cells Fig. 5d and e). We saw significant reduction of DTA-1 staining on CD8 T cells, and a trend of reduction on Tregs. These data indicate that while GITRL-FP could bind targets, the fusion protein alone could not delay TC-1 tumor growth or generate an antitumor immune response.

### Expansion of primed antigen-specific CD8 T cells

Vaccination has been shown to be an effective way to generate E7-specific responses that lead to the prevention and inhibition of tumor growth in the TC-1 tumor model [26]. To determine whether GITRL-FP may need primed antigen-specific T cells to drive an antitumor response, we generated E7-specific T cells by vaccinating naïve C57BL/6 mice with 10 μg of an E7 SLP in CpG (AddaVax). Mice were vaccinated on day 0 and concurrently treated IP with GITRL-FP at 1 mg/kg bi weekly for 3 doses. After 8 days, splenic CD8 and CD4 T cells were evaluated for antigen specificity with an E7 dextramer. No differences in CD4 and CD8 percentages relative to total lymphocytes were seen, but there was a large increase in E7-specific cells from mice treated with GITRL-FP (Additional file 3: Figure S2A-C).

**Fig. 6:**
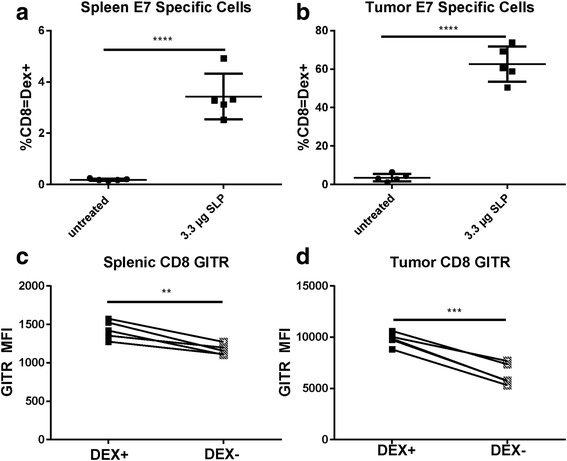
Generation of E7-specific GITR expression in TC-1 tumor‒bearing mice vaccinated with 10 μg of E7 SLP in CpG (Addavax) in the base of the tail. E7-specific CD8 T cells were evaluated in (**a**) spleens and (**b**) tumors. GITR levels were evaluated on E7 DEX+ cells and E7 DEX‒ CD8 T cells in (**c**) spleen and (**d**) tumor

In an evaluation of GITR levels on vaccinated mice alone, the GITR MFI of antigen-specific Dex^+^ cells was higher than that of Dex^–^ CD8 T cells (Fig. 6c). We hypothesized that this difference would be even higher in mice with tumors, as GITR levels seemed to be highest in the tumor microenvironment. To test this hypothesis, naïve C57BL/6 mice were implanted with TC-1 tumors and vaccinated with 10 μg of E7 SLP in CpG (AddaVax). Priming of E7 antigen-specific T cells was observed, as measured by dextramer in both spleens and tumors (Fig. 6a and b). Cells that stained positive with the dextramer were also present in higher numbers in both spleens and tumors (Fig. 6c-d), indicating that E7 SLP successfully primed an E7-specific response with or without the presence of tumor. Primed antigen-specific cells expressed higher levels of GITR than did other CD8 T cells.

### GITRL-FP agonism of GITR and mediation of PD effects in TC-1 tumors

**Fig. 7:**
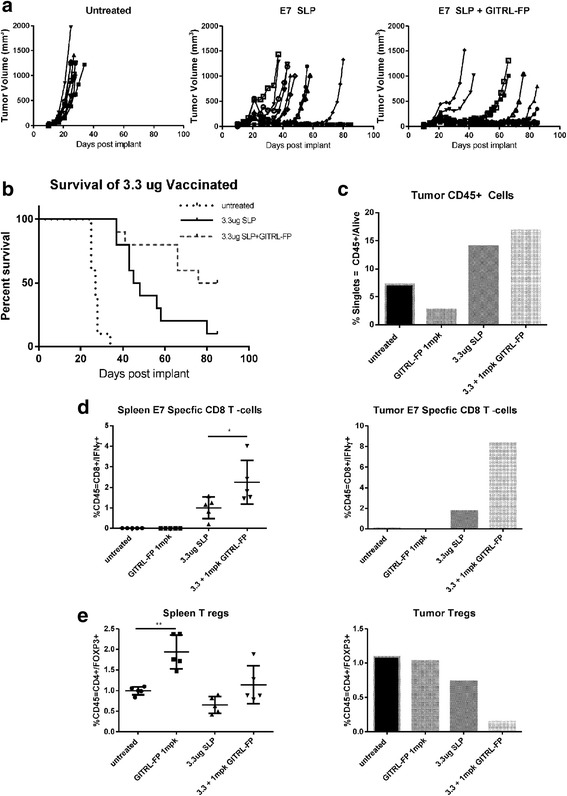
GITRL-FP agonism of GITR and mediation of PD effects in TC-1 tumors. **a** Tumor volumes were measured in TC-1 tumor–bearing mice vaccinated with vehicle or 3.3 μg of E7 SLP in CpG (Addavax) in the base of the tail. Treated mice received IP doses of GITRL-FP biweekly for 4 total doses. **b** Kaplan-Meier survival plot of TC-1 tumor bearing mice after treatment with E7 SLP and GITRL-FP To examine PD effects, we evaluated separate groups of mice with identical treatments. Tumors were pooled due to low cell numbers, and spleens were analyzed individually. **c** CD45^+^ cells in tumors. **d** Mouse spleens and tumors were restimulated with 1 μg/mL E7 peptide/protein transport inhibitor for 5 h and stained for IFNγ and TNFα (**e**) Tregs

To evaluate whether GITRL-FP could expand an E7-specific response to TC-1 tumor cells, C57BL/6 mice were implanted with TC-1 cells. On day 14 mice with tumor volumes of 0.035 to 0.085 cm^3^ were randomized into untreated control groups and treatment groups. Treated mice received single IP doses of E7 SLP with CpG (AddaVax) and GITRL-FP biweekly for a total of 4 doses, starting on the day of vaccination (Fig 7a). The study comprised 2 arms, a TGI group (*n* = 10) and a PD group (*n* = 5). Control mice had rapid tumor growth, and all died; median survival was 27 days. In mice implanted with TC-1 tumors and vaccinated with E7 SLP, 1 of 10 were alive and tumor free at day 85; median survival was 46.5 days. E7 SLP vaccination combined with GITRL-FP treatment resulted in significantly delayed tumor growth: 3 of 10 mice were tumor free and 5 of 10 were alive at day 85, with a median survival of 80.5 days [*P*-value = 0.0328; Mantel-Cox] (Fig. 7b and Additional file 4: Table S2). Vaccination alone resulted in delayed tumor progression, and adding GITRL-FP further delayed tumor progression. On day 21, spleens and tumors of PD mice were harvested for PD analysis. Footpad tumors were pooled due to the small amount of tissue harvested. Vaccination alone or in combination with GITRL-FP resulted in a significant increase in CD45^+^ cells in tumors (Fig. 7c). To evaluate antigen-specific function, we restimulated single-cell suspensions of tumor and spleen cells with E7 peptide at 1 μg/mL, pooling tumors due to cell number and analyzing spleens individually. Vaccination provided a basal increase in antigen-specific cells, and the addition of GITRL-FP further increased levels in both spleens and tumors (Fig. 7d). Interestingly, GITRL-FP did not deplete spleen Tregs and depleted tumor Tregs only when E7 SLP vaccine was present (Fig. 7e).

## Discussion

GITR agonist antibodies have been shown to be a potent method of generating an antitumor response, and several groups have shown that GITR agonism causes regression and elimination of CT26 tumors in BALB/c mice [8, 10]. In our study, low levels of GITRL-FP produced an equally potent effect that was dependent on the presence of CD8 T cells. As shown here and elsewhere, CD8 T cells in tumors express high levels of GITR [11]. GITRL-FP can directly produce GITR agonism in vivo. Our results showed a significant proliferation of antigen-specific CD8 T cells that secreted TNFα and IFNγ. This increase in antigen-specific cells was maintained and led to long-lasting tumor immunity. Interestingly, GITRL-FP seemed to modulate the size of this memory pool in a dose-dependent manner. GITR has been shown to control both clonal expansion of viral-specific CD8 T cells and generation of long-term memory [15, 27]. In the self-priming CT26 tumor model described here, GITRL-FP expanded and maintained a long-lasting antitumor memory response. In a previous study of viral models, GITR has been shown to lead to expansion of antigen-specific cells by modulating survival rather than proliferation [14]. We observed significant KI-67 expression on total CD8 T cells exposed to GITRL-FP but no change in the percentage of CD8 T cells in tumors. Yet we observed a significant expansion of antigen-specific T cells, which may support the hypothesis that engagement with GITRL-FP leads to better survival of cells that would normally die. Further studies are needed to examine the exact mechanism of this expansion.

On the basis of our CT26 findings, we next investigated how GITRL-FP could modulate a mouse tumor system that did not self-prime to a tumor-associated antigen. We chose the TC-1 tumor model because it has been extensively used to study the effects of DNA, peptide, and virus-based tumor vaccines [22, 24, 28]. First we investigated whether GITRL-FP alone could generate an E7-specific response in mice with TC-1 tumors and observed no generation of antigen-specific CD8 T cells. T cells in tumors and the periphery did express GITR, but there was no delay in tumor growth. To evaluate these effects in the presence of antigen-specific cells, we explored an SLP vaccine of E7, the addition of which produced robust generation of the antigen-specific compartment in both naïve and TC-1 tumor–bearing mice. E7-SLP treatment also produced tumor regression and delayed tumor outgrowth, and the addition of GITRLP-FP to E7-SLP produced durable antitumor activity. These results are similar to those of other studies in which DTA-1 has been combined with an adenovirus E7 vaccine [29]. In addition to inhibiting tumor growth, GITRL-FP also further increased the percentage of E7-specific CD8 T cells in this study. Although this observation differs from those of previous reports on adenovirus E7, it could be due to differences in either the vaccine or the GITR agonist. Our data suggest that GITRL-FP works in the TC-1 model only when combined with a vaccine, likely because primed CD8 T cells are necessary to mount an effective antitumor response. To confirm this, one could combine GITRL-FP with adoptive transfer of low numbers of E7 reactive CD8 T cells.

Several groups have shown that GITR agonism causes the regression and elimination of CT26 tumors in BALB/c mice [8, 10]. To our knowledge, no other groups have looked at GITR expression directly on antigen-specific CD8 T cells. Our data, combined with that of others’, indicate that primed, tumor-specific CD8 T cells are critical for a GITR agonist to generate a durable antitumor response. Such a response can be accomplished by combining GITR agonism with vaccinations such as peptide pulsed cells, dendritic cell–based vaccines, or viral vectors [29–31]. Some tumors, such as CT26 and B16/F10, carry immunogenic antigens that can prime the de novo response of CD8 T cells, which GITR agonism can magnify in the right context [18]. We believe that this GITR-driven increase in antigen-specific CD8 T cells is due to greater GITR expression on those T cells.

These data provide several hypotheses worth exploring in human clinical trials including evaluating if GITRL-FP can expand human antigen specific CD8 T cells and generate long term memory. While this study looks at the CD8 T-cell responses in two murine tumor models, one limitation of this study is that the multimerization of GITR and GITRL may be different between human and mice. Additionally, different dosing regimens were not evaluated and will be necessary to further understand the effects of GITRL-FP on T-cell expansion. This and other important aspects can be further evaluated and validated in human clinical trials, such as the formation of anti-drug antibodies.

## Conclusions

In conclusion, our data show GITRL-FP can significantly expand antigen-specific CD8 T cells and lead to anti-tumor immunologic memory in two separate murine models. In a recent article review of GITR in the context of immune oncology [4], a number of clinical trials are currently evaluating GITR agonists for the treatment of a wide range of tumor indications. The data presented here are important and relevant because they suggest that GITR agonism may be most successful in indications with primed CD8 T cells which express higher levels of GITR, including tumors with high mutational load and in the presence of foreign antigens, such as virus-driven tumors. Without a primed response, specific combinations with a vaccine or other CD8 T-cell priming therapies may be necessary to derive maximum benefit from GITR agonists such as GITRL-FP.

## Additional files

Additional file 1: Table S1. Median survival of mice from CD8 depletion. (DOCX 12 kb)

Additional file 2: Figure S1. GITR expression in untreated mice with CT26 tumors. (A) GITR expression was evaluated on CD8 T cells and Tregs in spleens and tumors. After treatment with GITRL-FP, GITR expression was evaluated on (B) CD8 cells and (C) Tregs in spleens, and tumors. (TIF 78 kb)

Additional file 3: Figure S2. Antigen specificity in CD8 and CD4 T cells of C57BL/6 mice vaccinated with 10 μg of E7 SLP in CpG (Addavax) in the base of the tail. Mice were treated with GITRL-FP at 1 mg/kg for 3 doses, and spleens were evaluated for (A) CD8 T cells, (B) CD4 T cells, (C) E7 dextramer^+^ T-cells, (D) Tregs, and (E) GITR levels on E7 DEX^+^ cells and E7 DEX^–^ CD8 T cells. (TIF 251 kb)

Additional file 4: Table S2. Median survival of mice vaccinated with E7 SLP and treated with GITRL-FP*. (DOCX 12 kb)

## Abbreviations

Addavax: Squalene-based oil-in-water nano-emulsion
CD45+ cells: Leukocytes
CD8+ cells: Cytotoxic T cells
CpG: Unmethylated CpG synthetic oligonucleotide
CT26: Murine colon carcinoma line
E7: Transforming protein from HPV16
FOXP3+: Forkhead box P3
GITR: Glucocorticoid-induced tumor necrosis factor receptor
GITRL: Glucocorticoid-induced tumor necrosis factor receptor ligand
GITRL-FP: Multimeric fusion protein of GITRL
IFNγ: Interferon gamma
SLP: Synthetic Long Peptide
TC-1: Murine tumor line producing E6 and E7 from HPV16
TNFα: Tumor necrosis factor Alpha
Treg cells: FOXP3+ T regulatory Cells
