# Supplementary material for: GITR ligand fusion protein agonist enhances the tumor antigen–specific CD8 T-cell response and leads to long-lasting memory
Source: J Immunother Cancer. 2017 Jun 20;5:47. doi: 10.1186/s40425-017-0247-0 (PMC5477245; doi:10.1186/s40425-017-0247-0)
Supplement: Supplementary file 1 — Median survival of mice from CD8 depletion. (DOCX 12 kb) [file 40425_2017_247_MOESM1_ESM.docx]

| CD8 Depletion Group | Dose (mg/kg) | Median Survival (days) |
| --- | --- | --- |
| Isotype | 25 | 24 |
| DTA-1 | 5 | 42 |
| DTA-1 | 25 | 48 |
| GITRL-FP | 1 | 27 |

**Additional file 1: Table S1.** Median survival of mice from CD8 depletion
