# Supplementary figures and images for: GITR ligand fusion protein agonist enhances the tumor antigen–specific CD8 T-cell response and leads to long-lasting memory
Source: J Immunother Cancer. 2017 Jun 20;5:47. doi: 10.1186/s40425-017-0247-0 (PMC5477245; doi:10.1186/s40425-017-0247-0)

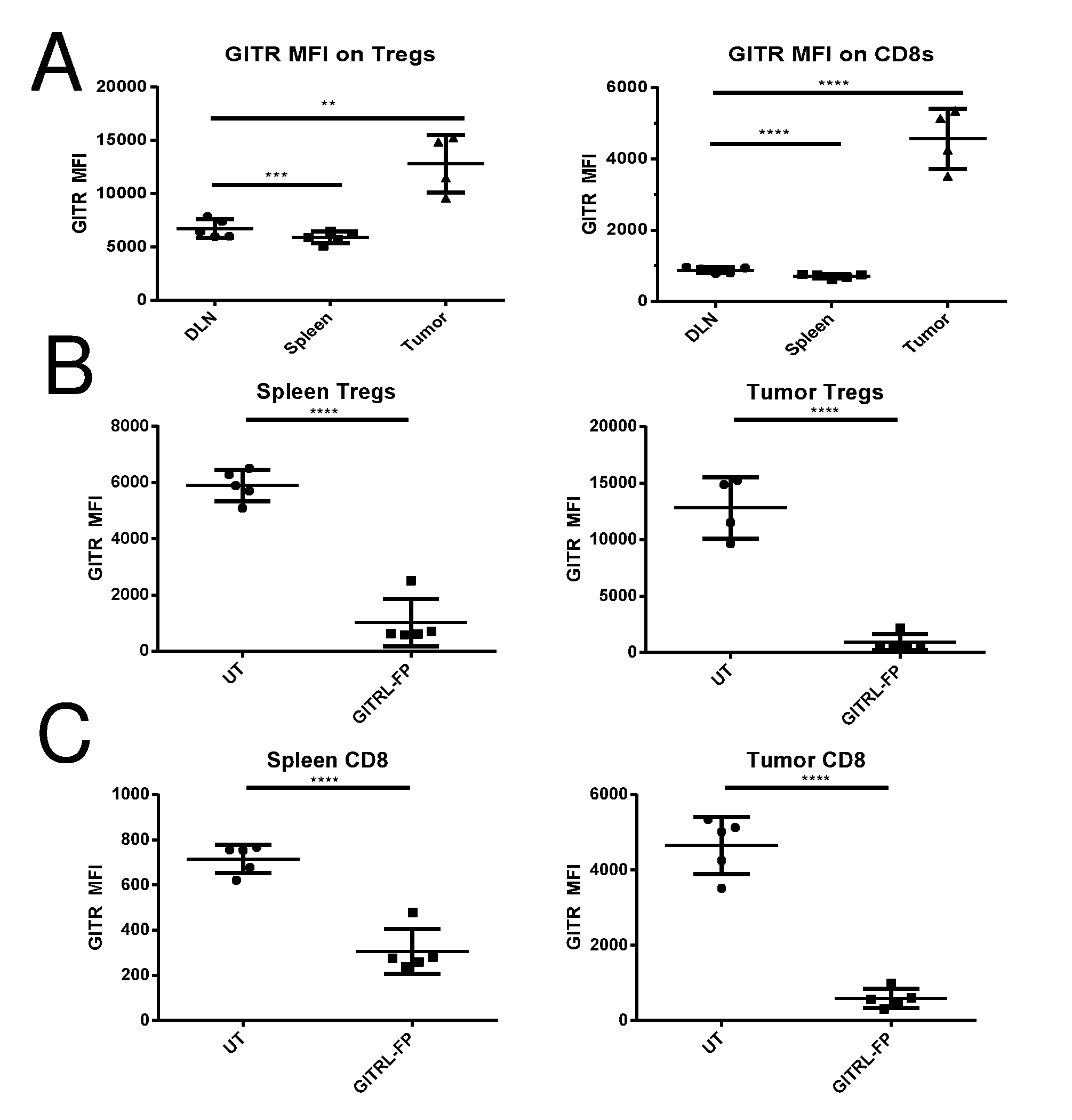

Supplement: Supplementary file 2 — GITR expression in untreated mice with CT26 tumors. (A) GITR expression was evaluated on CD8 T cells and Tregs in spleens and tumors. After treatment with GITRL-FP, GITR expression was evaluated on (B) CD8 cells and (C) Tregs in spleens, and tumors. (TIF 78 kb) [file 40425_2017_247_MOESM2_ESM.tif]

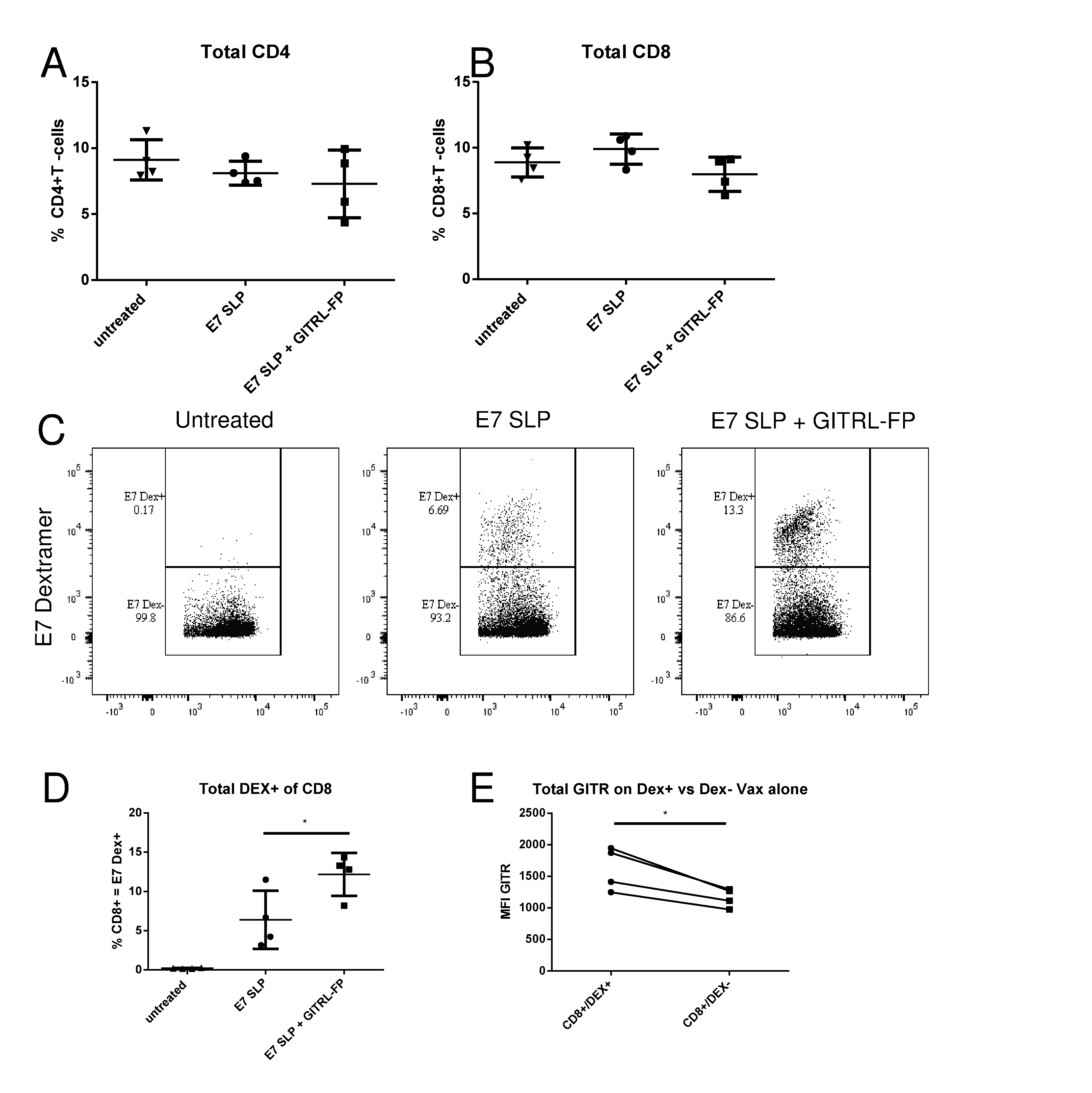

Supplement: Supplementary file 3 — Antigen specificity in CD8 and CD4 T cells of C57BL/6 mice vaccinated with 10 μg of E7 SLP in CpG (Addavax) in the base of the tail. Mice were treated with GITRL-FP at 1 mg/kg for 3 doses, and spleens were evaluated for (A) CD8 T cells, (B) CD4 T cells, (C) E7 dextramer+ T-cells, (D) Tregs, and (E) GITR levels on E7 DEX+ cells and E7 DEX– CD8 T cells. (TIF 251 kb) [file 40425_2017_247_MOESM3_ESM.tif]
