# Supplementary material for: GITR ligand fusion protein agonist enhances the tumor antigen–specific CD8 T-cell response and leads to long-lasting memory
Source: J Immunother Cancer. 2017 Jun 20;5:47. doi: 10.1186/s40425-017-0247-0 (PMC5477245; doi:10.1186/s40425-017-0247-0)
Supplement: Supplementary file 4 — Median survival of mice vaccinated with E7 SLP and treated with GITRL-FP*. (DOCX 12 kb) [file 40425_2017_247_MOESM4_ESM.docx]

**Additional file 4: Table S2.** Median survival of mice vaccinated with E7 SLP and treated with GITRL-FP*

|  | Group |  | Median Survival (days) |  |
| --- | --- | --- | --- | --- |
|  | Untreated |  | 27.0 |  |
|  | E7 SLP alone |  | 46.5 |  |
|  | E7 SLP + GITRL-FP(1) |  | 80.5 |  |
